# Clustering of cardiovascular risk factors in semi-urban communities in south-western Nigeria

**DOI:** 10.5830/CVJA-2016-024

**Published:** 2016

**Authors:** R Oluyombo, PO Akinwusi, MA Olamoyegun,, OE Ayodele, MB Fawale, OO Okunola, A Akinsola, TO Olanrewaju

**Affiliations:** Renal Unit, Department of Internal Medicine, Federal Teaching Hospital, Ido-Ekiti, Ekiti State, Nigeria; Cardiology Unit, College of Health Sciences, Osun State University, Osogbo, Nigeria; Department of Internal Medicine, Ladoke Akintola University of Technology Teaching Hospital, Ogbomoso, Oyo State, Nigeria; Department of Internal Medicine, Ladoke Akintola University of Technology Teaching Hospital, Ogbomoso, Oyo State, Nigeria; Department of Internal Medicine, Obafemi Awolowo University Teaching Hospitals, Ile-Ife, Osun State, Nigeria; Department of Internal Medicine, Obafemi Awolowo University Teaching Hospitals, Ile-Ife, Osun State, Nigeria; Department of Internal Medicine, Obafemi Awolowo University Teaching Hospitals, Ile-Ife, Osun State, Nigeria; Renal Division, Department of Medicine, University of Ilorin Teaching Hospital, Ilorin, Kwara State, Nigeria

**Keywords:** Clustering, cardiovascular, risk factors

## Abstract

**Background:**

In addition to poor socio-economic indices and a high prevalence of infectious diseases, there have been various reports of a rising prevalence of cardiovascular diseases, with associated morbidity and mortality in developing countries. These factors co-exist, resulting in a synergy, with serious complications, difficult-to-treat conditions and fatal outcomes. Hence this study was conducted to determine the clustering of cardiovascular disease risk factors and its pattern in semi-urban communities in south-western Nigeria.

**Methods:**

This was a cross sectional study over seven months in 11 semi-urban communities in south-western Nigeria.

**Results:**

The total number of participants was 1 285 but only 1 083, with 785 (65%) females, completed the data. Participants were 18 years and older, and 51.2% were over 60 years. The mean age was 55.12 ± 19.85 years. There were 2.6% current cigarette smokers, 22% drank alcohol and 12.2% added salt at the table, while 2% had been told by their doctors they had diabetes, and 23.6% had hypertension. The atherogenic index of plasma was at a high-risk level of 11.1%. Elevated total cholesterol and low-density lipoprotein cholesterol, and low high-density lipoprotein cholesterol levels were seen in 5.7, 3.7 and 65.1%, respectively. Prevalence of hypertension was 44.9%, diabetes was 5.2%, obesity with body mass index (BMI) > 30 kg/m^2^ was 5.7%, and abdominal circumference was 25.7%. Prevalence of clusters of two, three, and four or more risk factors was 23.1, 15.5 and 8.4%, respectively. Increasing age 2.94 (95% CI: 1.30–6.67), BMI 1.18 (95% CI: 1.02–1.37), fasting plasma glucose level 1.03 (95% CI: 1.00– 1.05), albuminuria 1.03 (95% CI: 1.00–1.05), systolic blood pressure 1.07 (95% CI: 1.04–1.10), diastolic blood pressure 1.06 (95% CI: 1.00–1.11) and female gender 2.94 (95% CI: 1.30–6.67) showed increased odds of clustering of two or more cardiovascular risk factors.

**Conclusion:**

Clustering of cardiovascular risk factors is prevalent in these communities. Patterns of clustering vary. This calls for aggressive and targeted public health interventions to prevent or reduce the burden of cardiovascular disease, as the consequences could be detrimental to the country.

## Background

Cardiovascular disease (CVD) is the leading cause of death globally, accounting for 17.3 million deaths per year. This is projected to increase to more than 23.6 million by 2030.^1^,^2^ It would be a crisis for developing countries to have to undergo this additional burden, as they are already faced with a multiple burden of other challenges, such as poor socio-economic indices, high prevalence of infectious diseases,^3^ and a trend towards highcaloric nutrition and sedentary lifestyles.^4^ An epidemic of CVD would have a detrimental effect on their already weakened health system.

In developing nations, unlike in developed countries, greater proportions of younger people are affected. Eighty per cent of deaths resulting from CVD occur between the ages of 30 and 70 years in developing countries.^5^ This is in contrast to 14 and 12% reported for the USA and UK, respectively.^6^ This would lead to depletion of the already insufficient workforce and a worsening of the poor economic status in developing nations. Nearly half of the annual output loss of US$ 500 billion is attributable to CVD.^7^

There have been reports of increased prevalence of CVD risk factors in Nigeria, with hypertension, diabetes, hyperlipidaemia and obesity as the leading modifiable causes.^8^,^9^ Studies have shown co-existence and interaction of these risk factors, causing them to become difficult-to-treat conditions, and resulting in serious complications and fatal outcomes.^10^,^11^. Findings from south-western and southern Nigerian people show a trend towards a high risk of developing major cardiovascular events over a 10-year period, with a cardiovascular mortality of 33.5% among individuals in the productive age group.^12^,^13^ Nigeria is the most populous country in Africa and has a population of 169 million, with over 50% living in rural communities.^3^

Surprisingly, in 2014, the World Health Organisation reported an absence of operational policies or action plans to reduce the risk factors for CVD. This is in spite of the goodwill adopted by world leaders at the United Nations General Assembly to reduce premature mortality from non-communicable diseases by 25% in 2025.^4^

Curtailing the challenge of CVD requires knowledge of its burden and risk factors, committed and effective socio-political interventions, and inexpensive strategies. This has contributed to the gradual and sustained decline in mortality in high-income countries.

This study therefore set out to determine the prevalence and pattern of clustering of risk factors, as this will effectively influence formulation of policies to curb detrimental health consequences in developing countries such as Nigeria. It was also of importance to determine whether clustering of cardiovascular risk factors occurs in patients from the semi-urban areas that access medical treatment from the hospitals where we practice, so we can subvert the imminent cardiovascular disease epidemic.

## Methods

This was a cross-sectional study spanning seven months, conducted in 11 semi-urban communities in Ekiti and Osun States, south-western Nigeria. Each of the towns was randomly picked from six local government areas (two communities per local government area). Using multi-staged sampling, participants aged 18 years and older were enrolled into the study.

In Osun state, Ilie in Olorunda local government was chosen. The local governments and towns chosen in Ekiti state were Ilejemeje (Iludun, Ilupeju), Ijero (Ayegunle, Oke-Iro), Ido-Osi (Ayetoro, Orin), Oye (Ilupeju, Itapa) and Moba (Osun, Ikun). In Ilie for instance, there were 32 compounds, out of which 16 were randomly selected. Out of these 16 compounds, eight were finally selected for the study. For some of the communities, convenient sampling was adopted for the peculiarities of these communities. The towns were predominantly agrarian with traders and few civil servants. Four hundred and sixty-eight and 835 participants were recruited from Osun and Ekiti states, respectively. Of the 1 285 enrolled in the study, 1 083 had complete data for analysis.

The community leaders had given prior consent after formal briefing in the presence of other chiefs who were the compound leaders. Informed consent was taken in the language best understood. The study was approved by the ethics committees of Ladoke Akintola University of Technology Teaching Hospital and Federal Medical centre, Ido-Ekiti.

Designated centres that were convenient for the subjects were used for the screening exercise. We used the World Health Organisation (WHO) STEPS questionnaires to obtain information from the participants.

## Sampling

Fasting blood samples (3 ml) were collected into lithium heparin bottles. Aseptic precautions were ensured. Fasting blood sugar was assayed with an Accu-check glucometer immediately after blood collection. The measuring range of the device for glucose is 50–600 mg/dl. Samples were thereafter taken to the chemical pathology laboratory of the Federal Medical Centre, Ido-Ekiti and Ladoke Akintola Teaching Hospital, Osogbo for analysis.

Samples were analysed for concentrations of total cholesterol (TC), high-density lipoprotein cholesterol (HDL-C), triglycerides (TG) and uric acid. For participants with TG values < 4.5 mmol/l, low-density lipoprotein cholesterol (LDL-C) was calculated from the Friedwald equation: LDL-C = (TC – HDL-C – TG)/5. Administration of questionnaires, biophysical measurements and collection of blood specimen was done by trained assistants who were also medical doctors.

Three measurements were taken after the participants had rested for five minutes. Participants were encouraged not to smoke, take alcohol or undertake exercise for at least 30 minutes before blood pressure measurement. Validated blood pressure apparatus (Omron M X2 Basic, Omron health care Co Ltd, Kyoto, Japan) was used. The average of two measurements was used if the difference between them was not more than 5 mmHg.

Height was measured to the nearest 0.1 cm using a standardised, marked measuring tape. Participants were asked to stand barefoot against a tape-marked vertical wall. Weight was measured to the nearest 0.1 kg using a standardised bathroom scale. Waist circumference was taken midway between the sub-costal margin and the iliac crest, to the nearest 0.1 cm.

## Definitions

Cardiovascular disease refers to a group of diseases involving the heart and blood vessels or the sequelae of poor blood supply due to a diseased vascular system. Hypertension was defined as blood pressure ≥ 140/90 mmHg or the use of antihypertensive medication(s). Diabetes was defined as a fasting plasma glucose level ≥ 7 mmol/l or a reported history of diabetes, or the use of glucose-lowering drugs

Dyslipidaemia was defined according to the Adult Treatment Panel (ATP) III guidelines^14^ as having one or more of the following factors present: TC ≥ 5.2 mmol/l, TG ≥ 1.7 mmol/l, HDL-C < 1.03 mmol/l in men and < 1.30 mmol/l in women, LDL-C ≥ 3.4 mmol/l, or a history of medication with lipidlowering drugs. High atherogenic index was defined as TC/ HDL-C ≥ 5.

Indices of abnormal fat distribution were also defined according to the ATP III guidelines as waist circumference (WC) ≥ 94 cm in men and ≥ 80 cm in women; and body mass index (BMI) ≥ 25 kg/m^2^ as overweight and ≥ 30 kg/m^2^ as obese. Participants with high blood pressure or symptomatic diabetes were referred to hospital for treatment and encouraged to go for follow up.

## Statistical analysis

Data analysis was done using SPSS version 20.0 (SPSS Inc, Chicago, Illinois, USA). The prevalence of each of the cardiovascular risk factors was determined and they are presented as frequencies and percentages. Continuous variables are presented as mean ± SD while categorical variables are presented as frequencies and percentages. The prevalence of risk factors in clusters of two, three or more was determined. Multivariate analysis of risk factors associated with two or more risk factors was carried out and the results are expressed as odds ratios with 95% confidence interval (CI). A significance level of p < 0.05 was used.

## Results

There were 785 (65%) females, mainly petty traders (41.4%) and farmers (28.1%). The mean age of participants was 55.12 ± 19.85 years (Table 1), with participants aged ≥ 60 years constituting 51.4%. Six hundred and twenty-three (51.6%) subjects had no formal education and only 8.3% had tertiary education. Eightyfour per cent earned less than N20 000 ($120 US) per month. We analysed the data of 1 083 participants.

**Table 1:** Demographic, clinical and laboratory parameters of the participants by gender

**	**	*Male*	*Female*	**	**
*Variables*	*n*	*(mean ± SD)*	*(mean ± SD)*	*Total*	*p-value*
Mean age (years)	1083	51.8 ± 21.4	57.0 ± 18.7	55.1 ± 19.9	< 0.01
BMI (kg/m^2^)	1083	22.7 ± 19.5	24.0 ± 22.3	23.6 ± 21.4	0.28
WC (cm)	1083	80.7 ± 9.9	84.5 ± 12.5	83.2 ± 11.8	< 0.01
TC (mmol/l)	1083	3.4 ± 1.1	3.4 ± 1.1	3.4 ± 1.1	0.8
LDL-C (mmol/l)	1083	1.5	1.6	1.6	< 0.01
HDL-C (mmol/l)	1083	1.0	1.0	1.0	0.5
TG (mmol/l)	1083	1.0	0.9	0.9	< 0.01
Serum uric acid (mg/dl)	1083	8.4	6.7	6.7	< 0.01
SBP (mmHg)	1083	136.0 ± 25.4	137.5 ± 27.4	137.0 ± 26.8	0.3
DBP (mmHg)	1083	78.1 ± 13.6	80.1 ± 13.3	79.4 ± 13.4	< 0.01
Urine ACR (mg/g)	754	15.0	20.0	20.0	0.5
FPG (mmol/l)	689	102.2 ± 30.5	98.2 ± 28.4	99.7 ± 29.3	0.08

BMI: body mass index, WC: waist circumference, TC: total cholesterol, LDL-C: low-density lipoprotein cholesterol, HDL-C: high-density lipoprotein cholesterol, TG: triglycerides, SBP: systolic blood pressure, DBP: diastolic blood pressure, ACR: albumin–creatinine ratio, FPG: fasting plasma glucose.

Two hundred and sixty-six (22%) subjects consumed alcohol, mainly beer (43.3%) and fresh palm wine (35.1%). Ninety-eight per cent added salt to their meals while cooking but only 12.2% added salt on the table while eating, and 43.5% were involved in vigorous activity that increased heart rate and breathing. Twenty-four (2%) participants had been told they had diabetes and 18 (75%) were receiving treatment from medical doctors, while seven (29%) used herbal remedies. There were 286 (23.6%) participants with a prior history of hypertension before the screening exercise.

Thirty-two (2.6%) participants were current cigarette smokers, 69 (5.7%) had elevated total cholesterol levels, 244 (20.2%) elevated triglyceride levels, and 69 (5.7%) were obese; 65.1% had low HDL-C values, while 3.7% (45) had high LDL-C levels and 11.1% high-risk atherogenic plasma index. Diabetes and elevated uric acid levels were present in 63 (5.2%) and 422 (34.9%) participants, respectively.

Systolic blood pressure (SBP) ≥ 140 mmHg and diastolic blood pressure (DBP) ≥ 90 mmHg were seen in 499 (41.3%) and 294 (22.4%), respectively. Two hundred and fifty (20.7%) participants had high systolic and diastolic blood pressures. Overall prevalence of hypertension was 542 (44.9%) subjects, of whom 383 (70.6%) were over 60 years of age.

Participants with two or more risk factors were older than those with none (p = 0.001) (Table 2), and similarly, the higher the mean values of waist circumference, the more the clustering of risk factors. There was a mean difference in SBP (14.6 ± 2.8 mmHg, p < 0.01) and DBP (6.3 ± 1.4 mmHg, p < 0.01), waist circumference (5.9 ± 1.2 cm, p < 0.01) and BMI (4.4 ± 2.2 kg/m^2^, p = 0.36) between participants with two risk factors and those with no risk factors (p < 0.01). However, a mean difference in BMI of 3.4 ± 0.5 kg/m^2^ was significant between subjects with three or more risk factors and those without any risk factors. At a mean difference of 0.34 ± 0.01 mmol/l, those with two or more risk factors had higher total cholesterol than those without risk factors (p = 0.006).

**Table 2:** Stratification of clustering of cardiovascular risk factors and mean values of selected risk factors among the participants

**	**	*No risk*	*1 risk*	*2 risk*	*3 risk*	*≥ 4 risk*	**
*Variables*	*n*	*factor*	*factor*	*factor*	*factors*	*factors*	*p-value*
Age (years)	1083	47.4 ± 21.3	49.9 ± 21.1	57.1 ± 19.4	64.8 ± 15.7	63.5 ± 14.0	< 0.01
WC (cm)	1067	77.5 ± 7.3	78.5 ± 8.4	83.5 ± 11.4	87.2 ± 12.6	94.2 ± 12.1	< 0.01
BMI (kg/m^2^)	1078	20.8 ± 2.1	21.3 ± 3.0	22.1 ± 3.9	24.2 ± 6.9	26.8 ± 5.8	< 0.01
SBP (mmHg)	1070	124.0 ± 20.4	128.2 ± 23.8	138.7 ± 26.3	151.1 ± 27.2	155.8 ± 26.4	< 0.01
DBP (mmHg)	1070	73.7 ± 10.5	75.4 ± 11.7	80.0 ± 13.2	85.4 ± 15.1	87.1 ± 11.6	< 0.01
TC (mmol/l)	1083	3.6 ± 0.9	3.3 ± 1.0	3.3 ± 1	3.4 ± 1.1	3.4 ± 1.5	0.04
LDL-C (mmol/l)	1083	1.5 ± 0.7	1.6 ± 0.8	1.6 ± 0.7	1.8 ± 0.9	1.9 ± 1.2	< 0.01
HDL-C (mmol/l)	1083	1.5 ± 0.6	1.0 ± 0.5	1 ± 0.4	0.9 ± 0.4	0.8 ± 0.4	< 0.01
TG (mmol/l)	1083	1.2 ± 0.9	1.2 ± 0.8	1.2 ± 0.8	1.1 ± 0.8	1.1 ± 0.8	0.58
TC/HDL-C	1083	2.8 ± 1.4	3.2 ± 2.5	3.3 ± 2.1	3.7 ± 2.5	4.1 ± 2.8	0.01
FBG (mmol/l)	608	5.0 ± 0.8	5.3 ± 0.9	5.4 ± 1.2	5.7 ± 2.1	6.4 ± 2.9	< 0.01

*Mean of the variables ± SD.

BMI: body mass index, SBP: systolic blood pressure, DBP: diastolic blood pressure, TC: total cholesterol, LDL-C: low-density lipoprotein cholesterol, HDL-C: high-density lipoprotein cholesterol, TC: total cholesterol, FPG: fasting plasma glucose.

**Fig 1.**
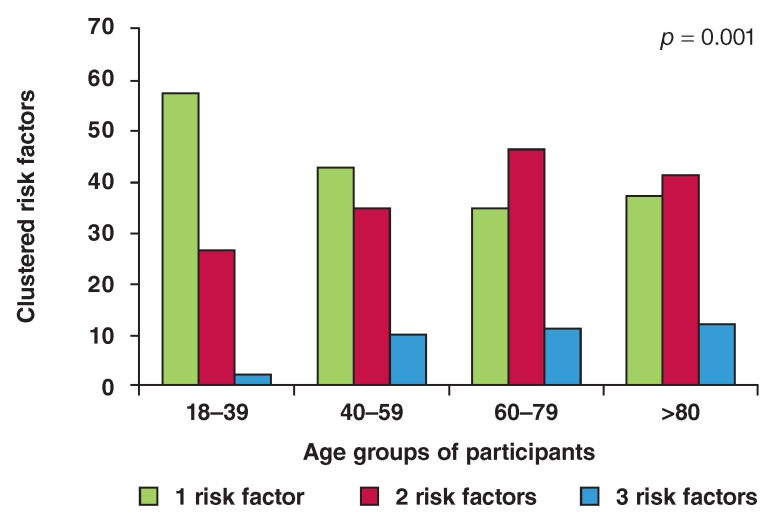
Age groups of participants and clustering of cardiovascular risk factors.

Classification by body adiposity showed that participants with overweight (BMI > 25 kg/m^2^) and obesity (BMI > 30 kg/m^2^) had higher clusters (two or more) of cardiovascular risk factors than those with normal weight (34.5 vs 49.3 vs 42%, p = 0.01). The prevalence of one, two and three or more cardiovascular risk factors were 35.7, 32 and 7.7%, respectively. Fig. 1 shows the stratified age distribution of prevalence of the clusters of risk factors. Prevalence of clusters of two, three, and four or more risk factors was 23.1, 15.5 and 8.4%, respectively.

**Fig 2.**
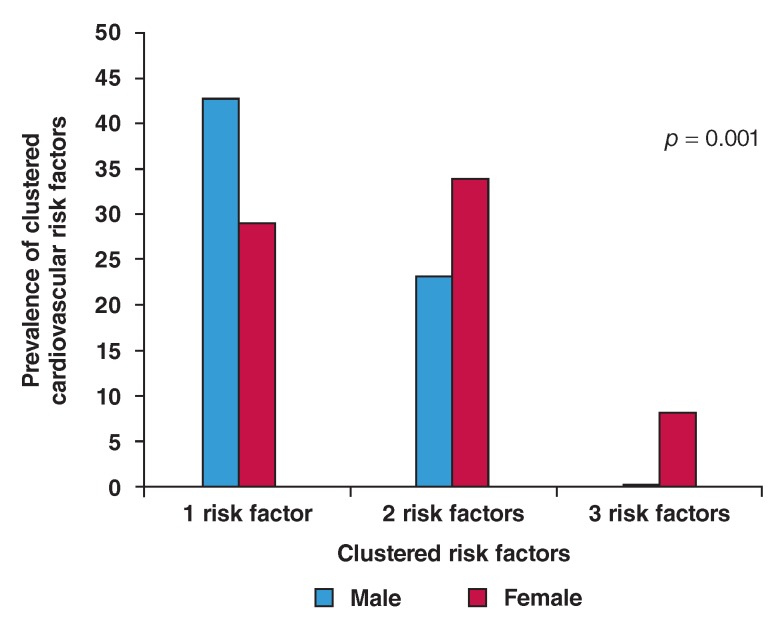
Distribution of cardiovascular risk factors cluster between men and women.

**Fig 3.**
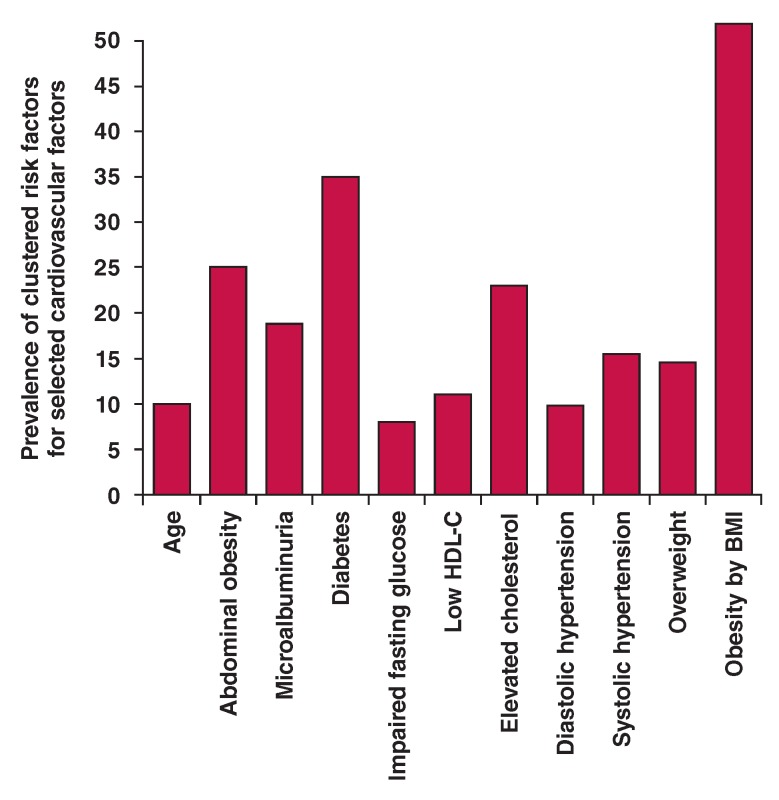
Selected cardiovascular risk factors and weight of clustering of other risk factors.

The number and burden of risk factors (cluster) increased with age (Table 2Fig. 2), and women had higher clustering of cardiovascular risk factors (p = 0.001) (Fig. 2). Selecting some risk factors, as shown in Fig. 4, participants with microalbuminuria had greater clusters of cardiovascular risk factors than those with normal values (21.2 vs 3.3%, p = 0.01). Similarly, as shown in Fig. 3, those with obesity (BMI > 30 kg/m^2^) (p = 0.001) and diabetes (p = 0.001) had more clusters (three or more) of cardiovascular risk factors.

Multivariate analysis (Fig. 3) between the selected risk factors and clustering of two or more risk factors showed increasing odds of clustering with increased age 1.07 (95% CI: 1.30–6.67), SBP 1.07 (95% CI: 1.04–1.10), DBP 1.06 (95% CI: 1.00–1.11) and BMI 1.18 (95% CI: 1.02–1.37).

## Discussion

This study has shown a high prevalence of cardiovascular risk factors and clustering of these risk factors among the study population. We found a prevalence of 32.9 and 8% of two and at least three cardiovascular risk clusters, respectively. Unlike in developed countries, but as seen in this study, the economically productive age groups were more affected.

The co-existence and synergistic effects of clusters of risk factors may explain the high burden and poor outcome of cardiovascular events such as stroke and death among blacks.^15^,^19^ The rapidity of lifestyle changes, increased market globalisation and the genetic make-up of the population could also explain this high prevalence. This is disturbing because the majority of people in the country have a low socio-economic status, with 84% earning N20 000 ($120) or less per month. The economic and social impact of cardiovascular disease would therefore be heavy on a developing country such as Nigeria if this trend is sustained.

With clustering of risk factors and increased clustering among the participants, the mean values of the risk factors were observed to be significantly related. There is a high rate of undiagnosed cardiovascular risk factors in Nigeria and the sub-region.^8^,^9^ The earlier the diagnosis is made the better the outcome, as this prevents progression to atherosclerosis, and worsening of non-conventional cardiovascular risk factors and associated end-organ damage, which is usually irreversible. This calls for regular screening and comprehensive examination of patients at every opportunity.

Studies have shown the impressive results of early intervention programmes.^20^ Lifestyle changes and/or the use of medications to treat hypertension, for instance, would reduce morbidity and mortality rates.^21^,^22^ Nowadays, diets that are rich in saturated fats and refined carbohydrates and low in vegetables, and increasing sedentary lifestyles are replacing traditional diets.

Males had a higher prevalence of a single risk factor, however, females had more clustering than males. This became more marked at middle age when clustering was more than doubled (Fig. 1). Our study showed significantly higher prevalence of low HDL-C, high LDL-C and triglyceride levels, obesity, and diastolic and systolic hypertension among women than men. These physiological mechanisms, in association with changes in their hormone levels with age, may be contributory.

In a similar community study conducted by Oladapo et al.,^9^ more women than men had a high prevalence of clustering of risk factors. More men than women had high blood pressure until 45 years of age but thereafter women caught up and later surpassed men in prevalence and occurrence of hypertension, coronary heart disease and stroke.^20^,^23^ Studies have also shown that females reported less physical activity than males.^24^,^25^

In this study, the higher the number of clustered risk factors, the higher the mean values of the risk factors. This suggests the need for appropriate preventative and therapeutic intervention to retard progression and prevent poor outcomes, with our limited health resources. Access to healthcare will increase utilisation of health facilities, provide early intervention through medication and lifestyle changes, and ensure regular monitoring. Policies on good dietary audits and healthy lifestyles should be developed and effectively implemented. Regular screening of populations at risk should also be encouraged.

There is evidence that therapeutic interventions are effective in treating overt medical conditions such as diabetes and hypertension, both of which in this study contributed significantly to clustering of risk factors.^26^,^27^ Similarly, obesity was associated with clustering of risk factors. This is in agreement with reports by Bayauli et al.^28^ in Congo and Dahiru^29^ in northern Nigeria.

The odds of clustering of cardiovascular risk factors increase with degree of obesity. In 2010, about 3.6 million deaths were estimated to result from overweight and obesity, with 3.9% years lost and 3.8% lost in disability-adjusted life-years.^30^ Prevalence of obesity has increased, not only in adults but also among children and adolescents in both developed and developing countries. Increased adiposity is a significant risk factor for atherogenesis and increased coagulability. Obesity is described as a chronic and systemic inflammatory disease as a result of the release of enormous pro-inflammatory cytokines and increasing insulin insensitivity. The rising prevalence of obesity is a threat to global health.

Microalbuminuria also increased the odds for cardiovascular risk clustering. Its presence suggests endothelial damage and it is an independent atherosclerotic risk factor.^31^ Its detection underscores high risk of cardiovascular disease and all-cause mortality, not just among people with diabetes but also in the general population.^32^,^33^ Prompt treatment of microalbumiuria among patients with diabetes, for instance, significantly ameliorates associated morbidity, such as diabetic nephropathy, which is usually a serious consequence. However, in view of the clustering of risk factors, multiple therapeutic approaches are suggested. This ensures coverage of most of the risk factors, as recommended in the guidelines.^34^,^35^

Varying reports have stressed the driving effect of hypertension and insulin resistance on other cardiovascular diseases. In this study, increasing blood pressure and plasma glucose levels were independently associated with increasing odds of clustering of risk factors. Few other studies have refuted the possible association, especially insulin resistance and other risk factors.

Our study demonstrated that each of the cardiovascular risk factors has varying degrees of clustering. The interplay among these various factors leads to similar physiological and structural dysfunction. For instance, microalbuminuria, insulin insensitivity and diabetes are associated with endothelial dysfunction.^36^ Sloten et al. therefore suggested therapeutic interventions that would target the common pathology and control risk factors that interact rather than those that do not interact.^37^

Our study has some limitations. It was a cross-sectional study. We were unable to discuss the sequence of events, and causality could not be established for cardiovascular events. The diagnoses of diabetes and hypertension were made during one visit, although protocols as recommended in the guidelines were strictly adhered to. Also, microalbuminuria was checked only once, as efforts to collect the samples after three months were frustrated by poor participation. On average, about one out of four initial participants re-presented for the second screening. This was terminated after the third community was visited, with the same experience.

## Conclusion

This study has shown not only the presence of cardiovascular risk factors, as in other studies, but also a high prevalence of clusters of such risk factors. The pattern of clustering showed significant association with conventional cardiovascular risk factors. These clusters will increase the health burden, promote rapid progression to end-organ damage and increased mortality rates if there is no planned and appropriate intervention. This is of great concern as it also portends a dwindling socioeconomic status in developing nations. It is important to stress a comprehensive approach of primary, secondary and tertiary preventative measures and control of these factors in order to reduce the overall burden of cardiovascular diseases.
